# Immobilization techniques improve volumetric hydrogen productivity of *Caldicellulosiruptor* species in a modified continuous stirred tank reactor

**DOI:** 10.1186/s13068-023-02273-8

**Published:** 2023-02-16

**Authors:** Thitiwut Vongkampang, Krishnan Sreenivas, Carl Grey, Ed W. J. van Niel

**Affiliations:** 1 Department of Applied Microbiology, Lund University, 124, 221 00 Lund, Sweden; 2 Department of Biotechnology, Lund University, 124, 221 00 Lund, Sweden; 3 Present Address: Biorefinery and Functional Food Research Unit, Department of Biotechnology, Faculty of Technology, Mahasarakham University, Kantharawichai, Mahasarakham 44150 Thailand

**Keywords:** *Caldicellulosiruptor kronotskyensis*, *Caldicellulosiruptor owensensis*, Acrylic fibres, Chitosan, Volumetric hydrogen productivity (Q_H2_)

## Abstract

**Background:**

Co-cultures and cell immobilization have been used for retaining biomass in a bioreactor, with the aim to improve the volumetric hydrogen productivity (Q_H2_). *Caldicellulosiruptor kronotskyensis* is a strong cellulolytic species that possesses tāpirin proteins for attaching on lignocellulosic materials. *C. owensensis* has its reputation as a biofilm former. It was investigated whether continuous co-cultures of these two species with different types of carriers can improve the Q_H2_.

**Results:**

Q_H2_ up to 30 ± 0.2 mmol L^−1^ h^−1^ was obtained during pure culture of *C*. *kronotskyensis* with combined acrylic fibres and chitosan. In addition, the yield of hydrogen was 2.95 ± 0.1 mol H_2_ mol^−1^ sugars at a dilution rate (*D*) of 0.3 h^−1^. However, the second-best Q_H2_ 26.4 ± 1.9 mmol L^−1^ h^−1^ and 25.4 ± 0.6 mmol L^−1^ h^−1^ were obtained with a co-culture of *C. kronotskyensis* and *C*. *owensensis* with acrylic fibres only and a pure culture of *C. kronotskyensis* with acrylic fibres, respectively. Interestingly, the population dynamics revealed that *C. kronotskyensis* was the dominant species in the biofilm fraction, whereas *C*. *owensensis* was the dominant species in the planktonic phase. The highest amount of c-di-GMP (260 ± 27.3 µM at a *D* of 0.2 h^−1^) were found with the co-culture of *C. kronotskyensis* and *C*. *owensensis* without a carrier. This could be due to *Caldicellulosiruptor* producing c-di-GMP as a second messenger for regulation of the biofilms under the high dilution rate (*D*) to prevent washout.

**Conclusions:**

The cell immobilization strategy using a combination of carriers exhibited a promising approach to enhance the Q_H2_. The Q_H2_ obtained during the continuous culture of *C. kronotskyensis* with combined acrylic fibres and chitosan gave the highest Q_H2_ among the pure culture and mixed cultures of *Caldicellulosiruptor* in the current study. Moreover, it was the highest Q_H2_ among all cultures of *Caldicellulosiruptor* species studied so far.

**Supplementary Information:**

The online version contains supplementary material available at 10.1186/s13068-023-02273-8.

## Introduction

Hydrogen gas (H_2_) has two very strong features as fuel. First, H_2_ has the highest energy content per mass of any practical fuel. Secondly, it does not release carbon dioxide (CO_2_) during combustion [1]. However, worldwide most H_2_ (96%) is produced from fossil-based resources, such as natural gas, coal, and oil [2]. Unfortunately, these resources are non-renewable and release massive amounts of greenhouse gases into the atmosphere [3]. The other 4% of H_2_ is generated through sustainable methods, such as water electrolysis, geothermal, and biomass [2]. This clearly shows that it is still challenging to produce H_2_ competitively from renewable resources and highlights the need for improving eco-friendly and sustainable methods, to make them economically feasible. Biological hydrogen production is a promising method using microorganisms to convert organic material to H_2_ [4]. Potential feedstocks are agriculture residues, food waste, and waste streams from industries and municipalities [2, 5].

The genus *Caldicellulosiruptor* has a good reputation for hydrogen production from a broad range of mono-, oligo- and polysaccharides such as maltose, mannose, xylan, cellobiose, and (hemi)cellulose [6]. Recently, a study on *C*. *kronotskyensis* revealed that this species produces tāpirin proteins, facilitating better attachment to lignocellulosic materials [7]. In addition, *C*. *kronotskyensis* was classified as strongly cellulolytic within its genus due to its possession of tapirin proteins and the number of cellulase-related glycoside hydrolases (GH) families [8]. In addition, several of its hydrolases have been investigated in dedicated studies, including xylanase [9] and pectate lyase [10] for degradation of plant biomass [11]. A recent study showed that *C*. *kronotskyensis* can be used for lignocellulosic degradation in a primary fermentation. Subsequently, *Cupriavidus necator* was employed to produce polyhydroxybutyrate (PHB) production through a sequential batch fermentation. Surprisingly, the production of PHB increased nine folds [12]. Moreover, a study of sugar uptake in *C*. *kronotskyensis* revealed that it possesses better uptake kinetics for xylose and cellobiose than for glucose and it showcased that it is a promising candidate for hydrogen production [13].

*C. owensensis* has been demonstrated to be a biofilm former [14] and like the other *Caldicellulosiruptor* species; it has the ability to metabolize a broad range of substrates [15]. The advantage of biofilm produced by *C. owensensis* that it retains biomass, thereby improving volumetric hydrogen productivity (Q_H2_) in both a CSTR and an Upflow Anaerobic bioreactor [16].

Bis-(3ʹ,5ʹ)-cyclic di-guanosine monophosphate (c-di-GMP) is a second messenger involved in the regulation between planktonic and a sessile lifestyle [17]. The regulation mechanism for c-di-GMP was found in both gram-negative [17] and gram-positive bacteria [18, 19]. Briefly, two molecules of guanosine triphosphate (GTP) are converted for the synthesis of one molecule of c-di-GMP by the enzyme diguanylate cyclase (DGC). On the other hand, phosphodiesterase (PDE) is used for hydrolysing c-di-GMP into 5'-phosphoguanylyl-(3'-5')-guanosine (pGpG) and guanosine monophosphate (GMP) [20, 21]. The enzyme DGC is produced from the GGDEF domain, whereas the enzyme PDE is synthesized by either the EAL or HD-GYP domain [21]. Similar to *Listeria monocytogenes* [18], *Caldicellulosiruptor* species carry both enzyme DGC and PDE [16, 22].

Chitosan is a biopolymer composed of the two monomers (β-1,4 linkages) 2-aceta-mido-2-deoxy-D-glucopyranose and 2-amino-2-deoxy-D-glucopyranose [23]. In general, this biopolymer is obtained by partial de-acetylation of chitin [24], resulting in a random distribution of the two monomers. Although chitosan has an antimicrobial property, a previous study established safe conditions for *Caldicellulosiruptor* species to enable chitosan to be used for cell immobilization [25]. However, immobilization with chitosan did not achieve the desired volumetric hydrogen productivity (Q_H2_). Nevertheless, a combination of chitosan with acrylic fibres as carriers led to stable biofilms, thereby improving Q_H2_ [25]. Acrylic fibres are linear polymer synthesized from polyacrylonitrile (PAN) (C_3_H_3_N)_n_ [26]. A previous study revealed that acrylic fibres exhibited suitable properties for enzyme immobilization [27, 28]. Moreover, acrylic fibres were used to immobilize purple non-sulphur bacteria for hydrogen production [29]. Therefore, we hypothesized that acrylic fibre has a high potential for immobilization of *Caldicellulosiruptor* species, useful in continuous cultures.

The aim of this study was to improve the volumetric hydrogen productivity (Q_H2_) through immobilization techniques using different types of carrier material applied in a CSTR. Co-cultures of *C*. *kronotskyensis* and *C. owensensis* were tested for possible enhanced immobilization leading to improve performance. In addition, the c-di-GMP levels related to biofilm formation during continuous cultures were also quantified.

## Results

### Q_H2_ and substrate consumption of*** C. kronotskyensis ***(Cases A–D)

**Fig. 1:**
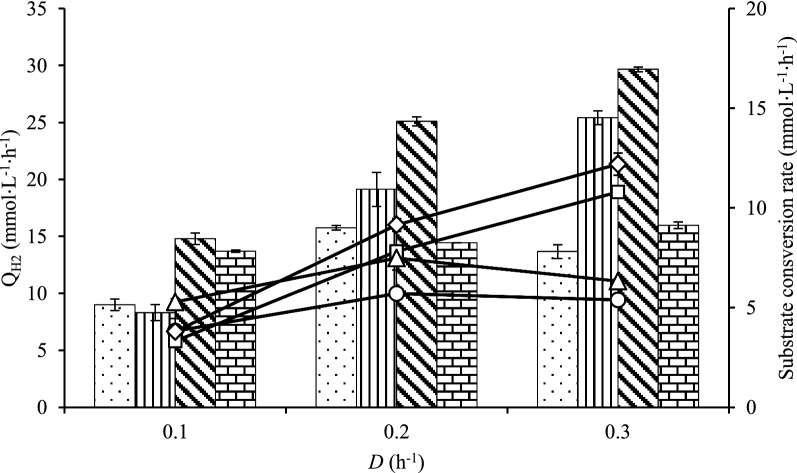
Volumetric hydrogen productivity (Q_H2_) and substrate consumption rate of pure culture of *C. kronotskyensis*. Q_H2_, bar graph (mmol L^−1^ h^−1^) and substrate consumption rate, line graph (mmol L^−1^ h^−1^). Case A (without carriers, dotted bar, open circle); Case B (with acrylic fibres, vertical line, open square); Case C (with acrylic fibres and chitosan, diagonal line, open diamond); and Case D (with chitosan, brick, open triangle)

The control *C. kronotskyensis* without carrier (Case A) showed improvement in the Q_H2_ at a dilution rate (*D*) up to 0.2 h^−1^, but then decreased at higher *D* (**Fig. ****1**). The Q_H2_ for *C. kronotskyensis* with acrylic fibres (Case B) increased notably from 8.3 to 25.4 mmol L^−1^ h^−1^ when the* D* was increased from 0.1 to 0.3 h^−1^. Similarly, the Q_H2_ for *C. kronotskyensis* with acrylic fibres and chitosan (Case C) was 30 mmol L^−1^ h^−1^. In contrast, no increase in Q_H2_ was observed with increasing *D* with chitosan only (Case D).

The substrate consumption rate (q_s_) for Case B was similar to Case C, displaying an increase of q_s_ with all the *D* (Fig. 1). In contrast, q_s_ for both Case A and Case D slightly increased when *D* was increased from 0.1 to 0.2 h^−1^ and did not change at the highest *D* studied.

### Q_H2_and substrate consumption of *** C. owensensis ***(Cases E–H)

The chemostat performance of *C. owensensis* without any carrier (Case E) ended as a washout without achieving a steady state at a *D* of 0.1 h^−1^ (Additional file 1: Table S1). The Q_H2_ of *C. owensensis* with acrylic fibres (Case F) and *C. owensensis* with acrylic fibres and chitosan (Case G) improved with the *D*. For *C. owensensis* with chitosan (Case H); the Q_H2_ increased between 0.1 and 0.2 h^−1^ and remained the level at a *D* of 0.3 h^−1^. The maximum Q_H2_ of Case F, Case G, and Case H were 20 mmol L^−1^ h^−1^, 19.5 mmol L^−1^ h^−1^, and 15.7 mmol L^−1^ h^−1^, respectively, at a *D* of 0.3 h^−1^.

**Fig. 2:**
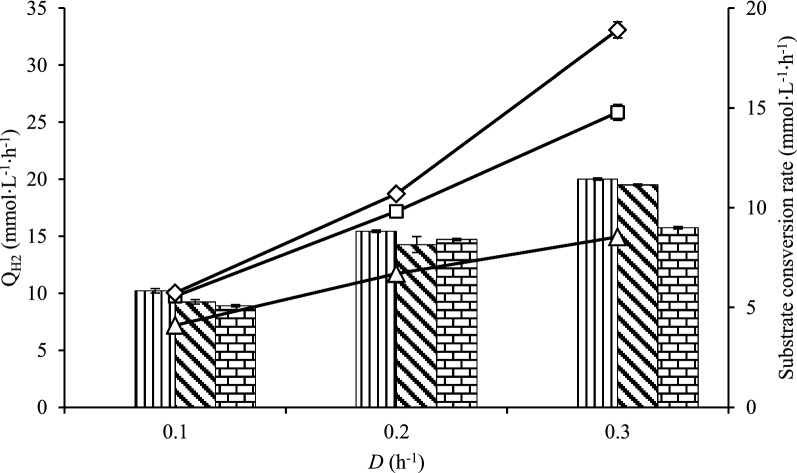
Volumetric hydrogen productivity (Q_H2_) and substrate consumption rate of pure culture of *C*. *owensensis*. Q_H2_, bar graph (mmol L^−1^ h^−1^) and substrate consumption rate, line graph (mmol L^−1^ h^−1^). Case F (with acrylic fibres, vertical line, open square); Case G (with acrylic fibres and chitosan, diagonal line, open diamond); and Case H (with chitosan, brick, open triangle)

The substrate consumption rate (q_s_) for Case F, Case G, and Case H displayed a similar pattern (Fig. 2). Notably, the highest q_s_ was obtained for Case G at a level of 18.9 mmol L^−1^ h^−1^ (at a *D* of 0.3 h^−1^) with the presence of acrylic fibres. In addition, the q_s_ for Case F was 14.8 mmol L^−1^ h^−1^ that is the second-best q_s_ at a *D* of 0.3 h^−1^.

### Q_H2_and substrate consumption of co-cultures (Cases I–L)

**Fig. 3:**
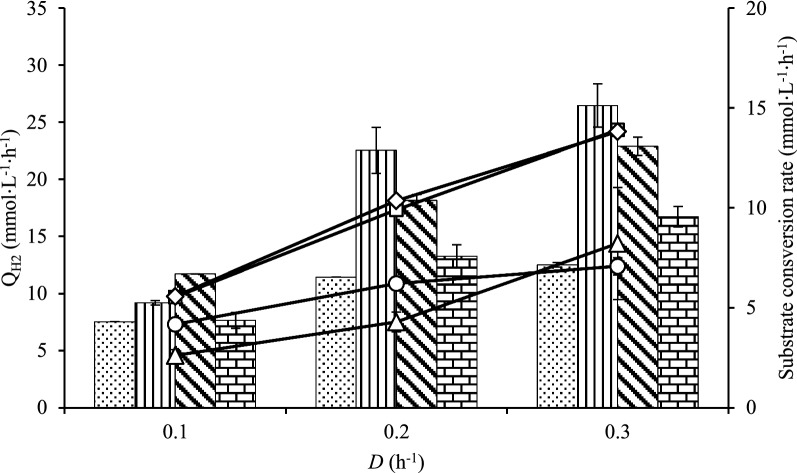
Volumetric hydrogen productivity (Q_H2_) and substrate consumption rate of co-culture of *C. kronotskyensis* and *C*. *owensensis*. Q_H2_, bar graph (mmol L^−1^ h^−1^) and substrate consumption rate, line graph (mmol L^−1^ h^−1^). Case I (without carriers, dotted bar, open circle); Case J (with acrylic fibres, vertical line, open square); Case K (with acrylic fibres and chitosan, diagonal line, open diamond); and Case L (with chitosan, brick, open triangle)

The chemostat of co-cultures of *C. kronotskyensis* and *C. owensensis* without carrier were performed (Case I, control study) (Fig. 3). The Q_H2_ of Case I increased from 7.5 to 11.5 mmol L^−1^ h^−1^ at a *D* of 0.1 h^−1^ and 0.2 h^−1^, respectively, and did not significantly further increase at a *D* of 0.3 h^−1^. Notably, the Q_H2_ of co-cultures with acrylic fibres (Case J), co-cultures with acrylic fibres and chitosan (Case K), and co-cultures with chitosan (Case L) improved when *D* was raised from 0.1 to 0.3 h^−1^. At a *D* of 0.3 h^−1^, the maximum Q_H2_ in Case J, Case K, and Case L were 26.5 mmol L^−1^ h^−1^, 23 mmol L^−1^ h^−1^, and 17 mmol L^−1^ h^−1^, respectively.

The q_s_ for both Cases J and K increased sharply with the *D* (Fig. 3) and for both cases it was similar (13.9 mmol L^−1^ h^−1^) at a *D* of 0.3 h^−1^. However, the q_s_ for Case I was seemingly constant between 0.2 h^−1^ and 0.3 h^−1^, whereas it increased in Case L.

### C-di-GMP related to biofilm formation

It was previously reported that *Caldicellulosiruptor* produced c-di-GMP correlated with biofilm formation in a defined co-culture in a chemostat [16]. In the current study, c-di-GMP was found with all fermentations but the concentration depended on the culture conditions.

**Fig. 4:**
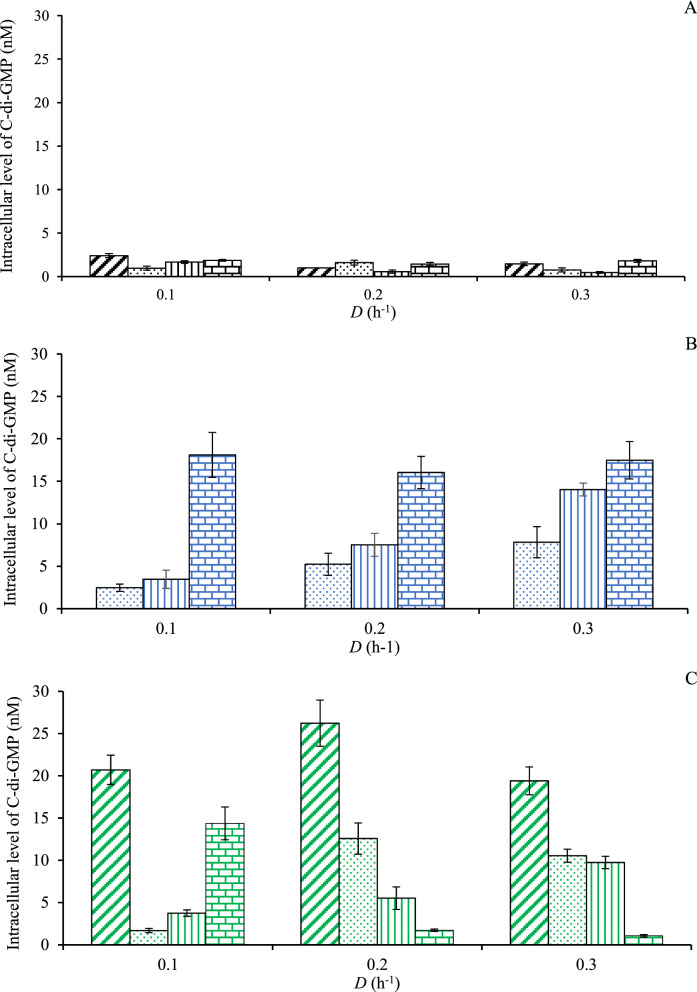
Intracellular level of c-di-GMP (µM) of pure cultures and co-cultures with and without carrier. **A** *C. kronotskyensis*: Case A (without carriers, diagonal line, black); Case B (with acrylic fibres, dotted, black); Case C** (**with acrylic fibres and chitosan, vertical line, black); and Case D (with chitosan, brick, black). **B** *C*. *owensensis*: Case F (with acrylic fibres, dotted, blue); Case G** (**with acrylic fibres and chitosan, vertical line, blue); and Case H (with chitosan, brick, blue). **C** Co-culture: Case I (without carriers, diagonal line, green); Case J (with acrylic fibres, dotted, green); Case K** (**with acrylic fibres and chitosan, vertical line, green); and Case L (with chitosan, brick, green)

The c-di-GMP levels for all pure cultures of *C. kronotskyensis* were very low in comparison to the other cultures studied (Fig. 4A). The average c-di-GMP concentrations were approximately between 0.5 and 2.4 nM and were not significantly affected by *D* or the presence of any of the carriers present.

On the other hand, the level of c-di-GMP in *C. owensensis* increased with *D* in the presence of acrylic fibres with and without chitosan (Cases F and G) (Fig. 4B). In contrast, c-di-GMP in Case H (with chitosan) showed similar levels at all *D*s, and importantly could reach levels up to 17.5 nM.

The highest c-di-GMP concentration of 26 nM was obtained during the co-culture of *C. kronotskyensis* and *C. owensensis* without carriers (Case I) at a *D* of 0.2 h^−1^ (Fig. 4C), whereas it was slightly lower (21 nM) at both a *D* of 0.1 h^−1^ and 0.3 h^−1^. In Case J, an amount of c-di-GMP increased approximately six-fold when the *D* was raised to 0.2 h^−1^ and 0.3 h^−1^. A gradual increase of c-di-GMP through all *D*’s was seen in Case K, when both acrylic fibres and chitosan were used for immobilization. However, with chitosan only (Case L) an amount of c-di-GMP drastically decreased from 14 nM at a *D* of 0.1 h^−1^ to 1.7 nM and 1 nM at a *D* of 0.2 h^−1^ and 0.3 h^−1^, respectively.

### Population dynamics of the co-cultures

The population dynamics of the co-cultures of *C. kronotskyensis* and *C. owensensis* were quantified during steady states at all dilution rates studied at the different conditions (Cases I–L).

#### Population dynamics of planktonic phase

**Fig. 5:**
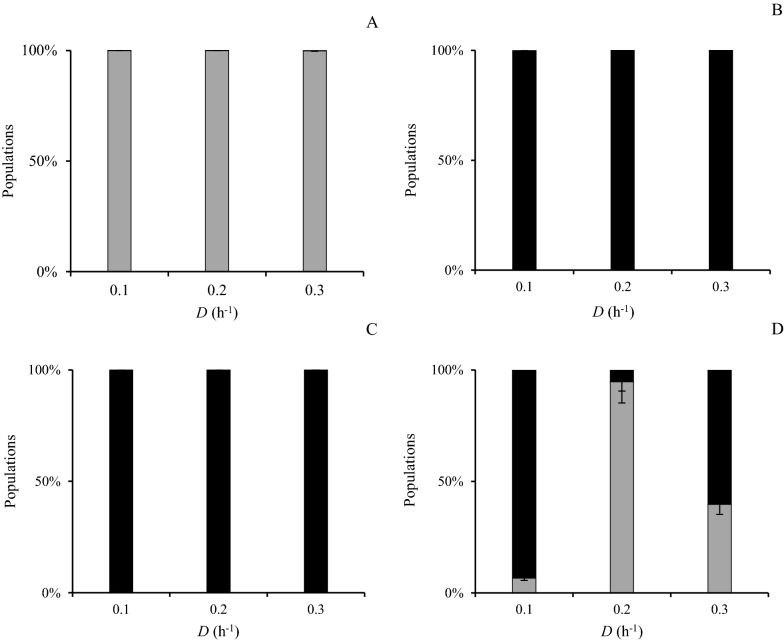
Fraction of *C. kronotskyensis* and *C*. *owensensis* in planktonic samples (Cases I–L). *C. kronotskyensis* (grey bar) and *C*. *owensensis* (black bar). **A** Co-culture without carrier (Case I). **B** Co-culture with acrylic fibres (Case J). **C** co-culture with acrylic fibres and chitosan (Case K). **D** Co-culture with chitosan (Case L)

The population dynamics of planktonic phase of the co-culture without carriers (Case I) indicated that *C. kronotskyensis* was the dominant species at all *D*’s (Fig. 5A), whereas *C. owensensis* was the superior species at all *D*’s in the cultivations in the presence of acrylic fibres with and without chitosan (Cases J and K) (Fig. 5B and C). In contrast to those three cases, the population dynamics of Case L (with chitosan) revealed that *C. owensensis* was the major species at a *D* of 0.1 h^−1^. Conversely, the population of *C. kronotskyensis* increased significantly to be the dominant species at a *D* of 0.2 h^−1^. Curiously, at a *D* of 0.3 h^−1^, the population of *C. kronotskyensis* dropped sharply to nearly half of the total population (Fig. 5D).

#### Population dynamics of biofilms phase

**Fig. 6:**
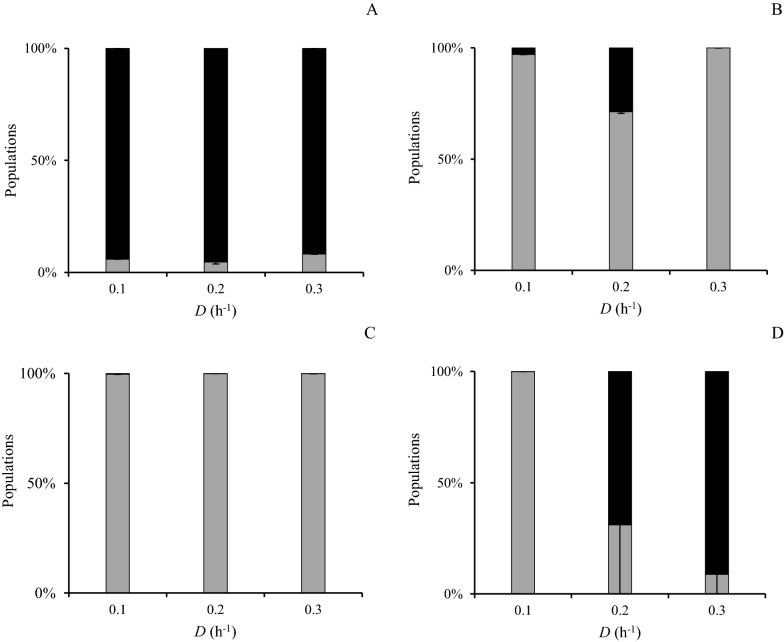
Fraction of *C. kronotskyensis* and *C*. *owensensis* in biofilm samples (Cases I–L). *C. kronotskyensis* (grey bar) and *C*. *owensensis* (black bar). **A** Co-culture without carrier (Case I). **B** Co-culture with acrylic fibres (Case J). **C** Co-culture with acrylic fibres and chitosan (Case K). **D** Co-culture with chitosan (Case L)

In Case I (co-culture without carriers), *C. owensensis* was the dominant species in the biofilms found on the glass and metal parts in the reactor at all the *D*s (Fig. 6A). Surprisingly, *C. kronotskyensis* was the dominant species in the presence of acrylic fibres with and without chitosan (Cases J and K) (Fig. 6B and C). In the presence of chitosan only (Case L) at a *D* of 0.1 h^−1^, *C. kronotskyensis* was the dominant species, but the population of *C. owensensis* increased significantly by seven-fold and nine-fold at the *D* of 0.2 h^−1^ to 0.3 h^−1^, respectively (Fig. 6D).

### Determination of yields, carbon balances, and redox balances

Product yields (Y) were calculated from the total amount of product formed per the net amount of sugar consumed. In this section, yields, carbon balances, and redox balances were only determined for continuous cultures at a *D* of 0.3 h^−1^. All these determinations were based on the samples of the planktonic part of the culture taken during each steady state.

#### Yield of pure cultures of *C. kronotskyensis*

**Fig. 7:**
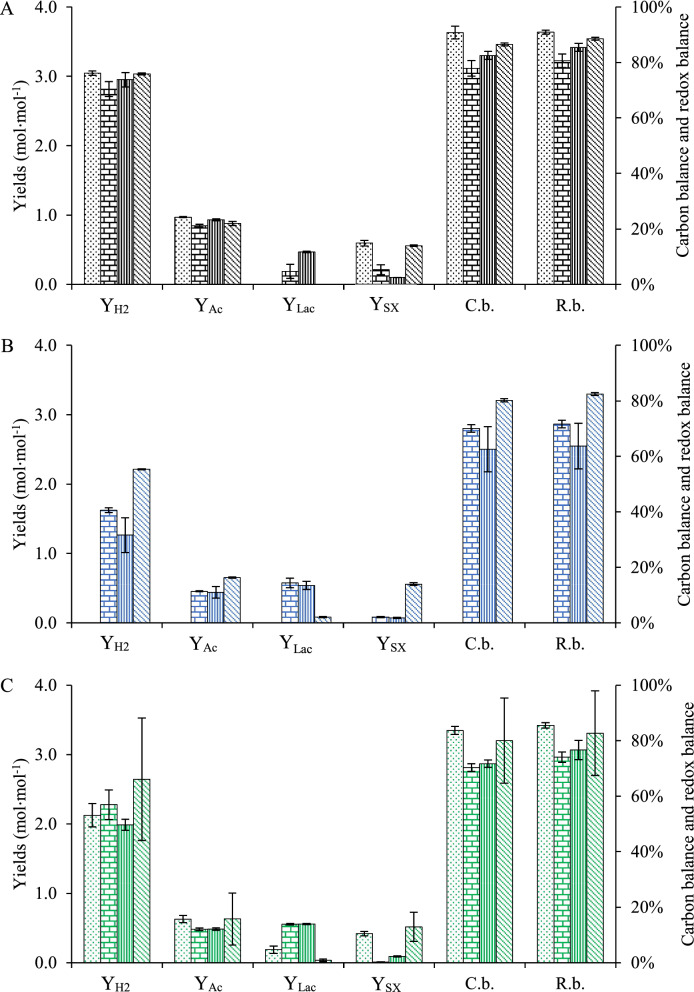
Yield coefficients, carbon balance (C.b.) and redox balance (R.b.) of all continuous cultures performed at a *D* of 0.3 h^−1^. **A** Case A (*C. kronotskyensis* without carriers, dotted bar, black); Case B (*C. kronotskyensis* with acrylic fibres, brick bar, black); Case C (*C. kronotskyensis* with acrylic fibres and chitosan, bar with vertical line, black); and Case D (*C. kronotskyensis* with chitosan, bar with diagonal line, black). **B** Case F (*C*. *owensensis* with acrylic fibres, brick bar, blue); Case G (*C*. *owensensis* with acrylic fibres and chitosan, bar with vertical line, blue); and Case H (*C*. *owensensis* with chitosan, bar with diagonal line, blue). **C** Case I (co-culture without carriers, dotted bar, green); Case J (co-culture with acrylic fibres, brick bar, green); Case K (co-culture with acrylic fibres and chitosan bar with vertical line, green); and Case L (co-culture with chitosan bar with diagonal line, green). Y_H2_, Y_Ac_, Y_Lac_, and Y_SX_ represent molar yields of H_2_, acetate, lactate, and biomass per mole of substrate consumed, respectively. C.b. and R.b. represent carbon balance and redox balance, respectively

For the pure culture of *C. kronotskyensis*, the yields of hydrogen (Y_H2_) and acetate yields (Y_Ac_) did not significant differ between the various conditions (Fig. 7A). The yield of lactate (Y_Lac_) for Case C (0.47 ± 0.01 mol^−1^ mol^−1^) was almost twice higher than that for Case B (0.19 ± 0.1 mol^−1^ mol^−1^), whereas no lactate formation for Case A and Case D was observed. The yield of biomass (Y_XS_) for Case A (0.59 ± 0.04 mol^−1^ mol^−1^) was similar to Case D (0.56 ± 0.01 mol^−1^ mol^−1^), and both were three folds and six folds higher than Case B (0.21 ± 0.07 mol^−1^ mol^−1^) and Case C (0.1 ± 0 mol^−1^ mol^−1^), respectively. The carbon balances (C.b.) and redox balances (R.b) of all cases were above 80%.

#### Yields of pure cultures of *C. owensensis*

As aforementioned, the pure culture of *C. owensensis* without carriers (Case E) was terminated due to washout. The Y_H2_ and Y_Ac_ for the culture of *C. owensensis* with chitosan (Case H) were almost 1.5 folds higher than the culture of *C. owensensis* with acrylic fibres (Case F) and of *C. owensensis* with acrylic fibres and chitosan (Case G) (Fig. 7B). In contrast, the Y_Lac_ for both Case F (0.58 ± 0.07 mol^−1^ mol^−1^) and Case G (0.54 ± 0.06 mol^−1^ mol^−1^) were six times higher than for Case H (0.08 ± 0.01 mol^−1^ mol^−1^). The Y_XS_ for Case H (0.56 ± 0.02 mol^−1^ mol^−1^) was seven folds higher than Case F (0.07 ± 0.01 mol^−1^ mol^−1^) and Case G (0.08 ± 0.01 mol^−1^ mol^−1^). The carbon balance (C.b.) and redox balance (R.b) for Case H were above 80%, whereas for both Case F and Case G they were 70%.

#### Yields of the co-cultures

The Y_H2_ and Y_Ac_ for all cases of co-cultures of *C. kronotskyensis* and *C*. *owensensis* with and without carriers were similar (Fig. 7C). The Y_Lac_ for co-culture with acrylic fibres (Case J; 0.55 ± 0.01 mol^−1^ mol^−1^) and co-culture with acrylic fibres and chitosan (Case K; 0.56 ± 0.01 mol^−1^ mol^−1^) were three folds and nearly twenty folds higher than the co-culture without carrier (Case I; 0.19 ± 0.05 mol^−1^ mol^−1^) and co-culture with chitosan (Case L; 0.03 ± 0.02 mol^−1^ mol^−1^), respectively. The Y_XS_ for Case I (0.42 ± 0.03 mol^−1^ mol^−1^) and Case L (0.52 ± 0.21 mol^−1^ mol^−1^) were five times and ten times higher than for Case K (0.09 ± 0.01 mol^−1^ mol^−1^) and Case J (0.01 ± 0 mol^−1^ mol^−1^), respectively. The carbon balance (C.b.) and redox balance (R.b) for Case I and Case K were above 80%, whereas those of both Case J and Case L were 70%.

## Discussions

The highest Q_H2_ in this study was obtained with a pure culture of *C. kronotskyensis* with acrylic fibres and chitosan as carriers (Case C), at a *D* of 0.3 h^−1^, being 30 mmol L^−1^ h^−1^. It was 1.5–3 folds higher than the Q_H2_ of the co-culture of *C*. *saccharolyticus* and *C*. *owensensis* in previous studies (Additional file 1: Table S2): (i) in an Anaerobic Upflow reactor with granular sludge (20 mmol L^−1^ h^−1^) [16], (ii) without carrier in trickle bed reactor [30], and (iii) in a fluidized bed system 10 mmol L^−1^ h^−1^ [14]. However, the Q_H2_ of thermophilic culture 33HL (45.8 mmol L^−1^ h^−1^) [31] was 1.5 times higher than the maximum Q_H2_ obtained in the current study (Case C; *C. kronotskyensis* with acrylic fibres and chitosan). The dominant bacteria in that study were *Thermobrachium celere* and *Thermoanaerobacterium aotearoense*.

In addition, the second-best Q_H2_ 25.4 ± 0.6 mmol L^−1^ h^−1^ and 26.4 ± 1.9 mmol L^−1^ h^−1^ were obtained with a pure culture of *C. kronotskyensis* with acrylic fibres (Case B) and a co-culture of *C. kronotskyensis* and *C*. *owensensis* with acrylic fibres only (Case J), respectively, both at a *D* of 0.3 h^−1^. Within the result for Case J, it can be argued that biofilms produced by *C. kronotskyensis* played a crucial role and similarly to the presence of chitosan used in Case C (*C. kronotskyensis* with acrylic fibres and chitosan) for retaining biomass in the CSTR. Moreover, at a *D* of 0.2 h^−1^, the Q_H2_ of Case J was at the same level as the Q_H2_ attained in Case C at the same *D* (Figs. 1 and 3). Furthermore, the substrate consumption rate (q_s_) of Case J was 13.9 mmol L^−1^ h^−1^ at a *D* of 0.3 h^−1^, which was slightly higher than the substrate consumption rate of Case C (12.2 mmol L^−1^ h^−1^ at a *D* of 0.3 h^−1^) (Figs. 1 and 3). The co-culture of *C. kronotskyensis* and *C*. *owensensis* with acrylic fibres and chitosan (Case K) was the third highest Q_H2_ (23 mmol L^−1^ h^−1^). It is worth noting that the presence of carrier(s) (either single one or combined) facilitates biofilm formation, thereby improving Q_H2_. Interestingly, the population analysis of Cases J and K revealed that *C. kronotskyensis* was the dominant species on acrylic fibres, whereas *C*. *owensensis* was the dominant species in planktonic phase (Fig. 6). We checked the biofilm visually and noticed that the cell mass in the biofilm was significantly higher than in the planktonic phase. Even though we did not quantify the biofilm, it indicated that *C. kronotskyensis* must have had the highest influenced on the Q_H2_ in both cases. In addition, the q_s_ in Cases J and K were both at the level of 13.9 mmol L^−1^ h^−1^ at a *D* of 0.3 h^−1^ (Fig. 3).

Without a carrier *C*. *owensensis* (Case E) washed out already before a *D* of 0.1 h^−1^, indicating that *C*. *owensensis* could not retain either planktonic cells or biofilms in a CSTR, even though *C*. *owensensis* is a biofilm former [14]. Therefore, insufficient data did not make it possible to calculate for the parameters shown in Figs. 1, 2, 3, 4, 5, 6, 7. In contrast, the pure culture of *C*. *owensensis* with acrylic fibres (Case F) and with acrylic fibres and chitosan (Case G) achieved a Q_H2_ at a level of approximately 20 mmol L^−1^ h^−1^, which was at similar level as described in a previous study [16].

In the current study, planktonic cells were collected for quantitative of c-di-GMP level. We assumed that their state also reflected that of the cells in the biofilm as the life cycle of biofilm is feed-and-bleed [16]. However, there is no straightforward pattern emerging from the co-culture analyses, as there appears to be contrasting results. This might be due to several unknowns. First, it may be possible that the presence of *C. owensensis* triggers c-di-GMP production in *C. kronotskyensis* (Fig. 4C) as the latter is dominant in the planktonic phase. Second, chitosan may be responsible for the stimulation in c-di GMP production by *C. owensensis* (Fig. 4B). And third, it might be possible that *C. kronotskyensis* cross feeding or stimulating *C. owensensis* so that the latter remains in the planktonic phase in the reactor (Fig. 5B and C). The c-di-GMP level of the co-culture of *C*. *kronotskyensis* and *C*. *owensensis* without a carrier (Case I) was 26 nM, being the highest c-di-GMP in this study. In contrast, the pure culture of *C*. *kronotskyensis* with and without carrier possessed the lowest levels (< 3 nM) (Fig. 4). A lower amount of c-di-GMP had been observed with the co-culture of *C*. *kronotskyensis* and *C*. *owensensis* with a single type of carrier and combined carriers (Fig. 4C). Thus, the levels were higher in the absence of proper carrier material, which might indicate the desire of cells to adhere, hence being in a state of transition to a biofilm lifestyle [17, 20, 21]. Interestingly, *C*. *owensensis* (Cases F, G, and H) possessed higher levels of c-di-GMP than *C*. *kronotskyensis* (Cases A, B, C, and D) in their pure cultures. This could be explained by the lifestyle of *C*. *owensensis* as biofilm former [14], and an amount of c-di-GMP of pure culture of *C*. *owensensis* obtained in this study were similar as seen in a previous study [16].

None of the c-di-GMP levels of pure culture of *C*. *kronotskyensis* (Cases A, B, C, and D) were beyond 3 nM (Fig. 4A), suggesting that *C. kronotskyensis* was not stimulated to form a biofilm when cultured alone with the media used. Under conditions mimicking the natural habitat of *C. kronotskyensis*, it may be a good biofilm producer. Nonetheless, it was reported that *C*. *kronotskyensis* possesses four loci responsible for Che-type system involving in the regulation of diguanylate cyclases (DGC) and phosphodiesterases (PDE) and flagella or pilus synthesis [22]. Moreover, there were six genes encoding for GGDEF domains (pfam00990) in* C*. *kronotskyensis* using Integrated Microbial Genomes database (IMG: https://img.jgi.doe.gov). In contrast, nine genes responsible for GGDEF domains (pfam00990) were found in *C*. *owensensis*. Furthermore, Che-type system has not been studied in *C*. *owensensis*; therefore, no conclusion in literature can be made about the c-di-GMP levels and mode of adhesion by *Caldicellulosiruptor* species.

The population dynamics analysis revealed that *C*. *owensensis* was the dominant species found in the biofilm state during the co-culture of *C*. *kronotskyensis* and *C*. *owensensis* without a carrier (Case I). Therefore, this result indicated that the most influential species was *C*. *owensensis*. Moreover, this phenomenon could be the stimulation of growth between *Caldicellulosiruptor* species that has been previously reported [33]. Interestingly, *C*. *kronotskyensis* was the dominant species for the biofilm fractions in the presence of carriers (Fig. 6B and C). Like the co-culture with combined acrylic fibres and chitosan (Case K), *C*. *kronotskyensis* was the dominant species and the c-di-GMP levels were less than 15 nM (Fig. 4C). In fact, the c-di-GMP levels ranged between 100 and 200 µM in the co-culture of *C*. *saccharolyticus* and *C*. *owensensis* in a previous study [16].

The lower amounts of biomass of the planktonic cells in the presence of acrylic fibres reflect that it is compensated by immobilized biomass, which is not possible to determine. The higher concentrations of lactate can also be related to the biofilm in the acrylic fibres, which contributed to lower hydrogen and acetate yields. This was especially visible with *C*. *owensensis* in the pure and co-culture, and it is known that the species can produce significant amounts of lactate [32].

The yield of hydrogen (Y_H2_) was high in all pure cultures of *C*. *kronotskyensis* of which the culture with acrylic fibres and chitosan (Case C) was the highest, i.e. 2.95 ± 0.1 mol H_2_ mol^−1^ hexose at a *D* of 0.3 h^−1^ (Fig. 7A). It is higher than the Y_H2_ for the co-culture of *C*. *saccharolyticus* and *C*. *kristjanssonii* (2.5 mol H_2_ mol hexose^−1^) [33] and cultivation of thermophilic culture 33HL (1.54 mol H_2_⋅mol hexose^−1^) [31] at a *D* of 0.3 h^−1^. Nonetheless, the Y_H2_ observed from the continuous culture of *C*. *saccharolyticus* at a *D* of 0.3 h^−1^ was 3.1 mol H_2_ mol hexose^−1^ [34]. In that respect, *C*. *kronotskyensis* appears to be superior in relation to both volumetric productivity and yields of hydrogen and it may not need *C*. *owensensis* for biofilm formation.

## Conclusion

The current study displayed that immobilization strategies using combined carrier could improve volumetric hydrogen productivities (Q_H2_) to a maximum of 30 mmol L^−1^ h^−1^ with a pure culture of *C*. *kronotskyensis* in continuous culture. The co-culture strategy of using *C. owensensis* obtained lower Q_H2_ from which it became clear that *C*. *kronotskyensis* had superior performance and biofilm formation. Cell immobilization using acrylic fibres together with or without chitosan has led to biofilm formation. Chitosan aggregates the cells and keep them mobile in the culture but may have facilitated biofilm formation in the presence of acrylic fibres. These might be key parameters to obtain high Q_H2_ in a CSTR. Further research is required for improving volumetric hydrogen productivity, even further for which* C*. *kronotskyensis* is the best candidate together with other reactor types to reach cost-effective Q_H2_ values.

## Materials and methods

### Microorganism and cultivation medium

*Caldicellulosiruptor kronotskyensis* (DSM 18902) and *Caldicellulosiruptor owensensis* (DSM 13100) were purchased from the Deutsche Sammlung von Mikroorganismen und Zellkulturen (DSMZ; Braunschweig, Germany). Stock cultures were prepared in a 250-mL serum flask containing 50 mL of modified DSM 640 [35] with 10 g/L of glucose as sole carbon source under strict anaerobic condition and incubated at 70 °C. After growth, cells were taken with syringe and needle for injection in 80% glycerol serum flask as described by Ref. [33].

### Bioreactor setup

All continuous cultures were performed in a 3-L jacketed glass bioreactor with a working volume of 1L. The pH was maintained at 6.9 ± 0.1 using 4 M NaOH through a base pump operated by an ADI 1025 Bio-Console and AD 1010 Bio-controller (Applikon, Schiedam, The Netherlands). The temperature was maintained at 70 ± 1 °C. Nitrogen gas was supplied continuously through the culture medium at the rate of 6 L/h. The sugar concentration in the feed medium was 7.3 g/L of glucose and 3.4 g/L of xylose without yeast extract as per [36]. The medium containing glucose and xylose mixtures were fed into the bioreactor with stepwise increase of the dilution rate (*D*), i.e. 0.1 h^−1^, 0.2 h^−1^, and 0.3 h^−1^. All the conditions performed in this study are given in Table 1.

**Table 1:** Fermentation conditions used in this study

Conditions		*C. kronotskyensis*	*C*. *owensensis*	Co-culture
No acrylic fibres	No chitosan	Case A	Case E	Case I
Acrylic fibres	No chitosan	Case B	Case F	Case J
Acrylic fibres	Chitosan	Case C	Case G	Case K
No acrylic fibres	Chitosan	Case D	Case H	Case L

### Quantitative analysis of H_2_ and CO_2_

Gas samples of 1 mL were taken from the bioreactor’s headspace to measure H_2_ and CO_2_ using Gas Chromatography (Agilent 7890B model, Santa Clara, CA, USA) connected with a TCD and a ShinCarbon ST 50/80 UM (2 m × 1/16 × 1 mm) column. The Gas Chromatography (GC) system was fed with helium gas through the column at a flow rate of 10 mL/min at 80 °C for 1 min, followed by increasing the temperature to 100 °C and holding for 4 min. Finally, the temperature was ramped and maintained at 160 °C for 2 min. The percentages of H_2_ and CO_2_ were calculated by the Agilent built-in software installed in the computer coupled with the GC. The validation of calibration curves for H_2_ and CO_2_ had been done after setting up of the GC.

The criteria for each steady state (between the *D* of 0.1 h^−1^ to 0.3 h^−1^) was determined the amount of hydrogen by using Gas Chromatography. The results should not excess over 5% comparing with the consecutive percentage of hydrogen. Therefore, the time point for taking samples at the specific steady state of each condition is different due to the addition of carrier that affected the state of biofilm formation, e.g. the steady state of Case A was taken at a *D* of 0.1 h^−1^ after 149 h (Additional file 1: Table S3). Moreover, all gas samples at specific steady state were taken in duplicate (Additional file 1: Table S3).

### Analytical method for HPLC

Liquid samples were withdrawn and centrifuged to obtain the supernatants, which were further analysed the concentration of glucose, xylose, acetate, lactate, ethanol, and propionate by an Aminex HPX-87H ion-exchange column (7.8 × 300 mm, Bio-Rad, Hercules, USA) facilitated in a high-performance liquid chromatography (HPLC) (Waters, Milford, MA, USA) connected with a refractive index detector (Shimadzu, Tokyo, Japan). H_2_SO_4_ (5 mM) was used as a mobile phase at a flow rate of 600 µL/min. The column was maintained at a temperature of 60 °C.

The data from HPLC were collected and used for the calculation of carbon balance.

1 $$\mathrm{Carbon\, balance}=\left(\frac{{C}_{out}}{{C}_{in}}\right)\times 100,$$

where *c*_*out*_ is the total amount of carbon measured from both HPLC and GC, and *c*_*in*_ is the total amount of carbon obtained in cultured medium.

Moreover, redox balance is the calculation of available electron, which can be seen in Eq. (2):

2 $$\mathrm{Redox\, balance}=\left(\frac{{e}_{out}}{{e}_{in}}\right)\times 100,$$

where *e*_*out*_ is the total electron in effluent and *e*_*in*_ is the total electron obtained in cultured medium.

### Determination of biomass

The optical density (OD) of the planktonic cells was determined at 620 nm using an Ultrospec 2100 pro UV/visible spectrophotometer (Amersham Biosciences, UK). Culture medium samples of 20 mL were withdrawn for the estimation of cell dry weight (CDW) using pre-weighed Supor-200, 0.2 µm filter (PALL Life Sciences, Mexico). The dry filters were weighed on an analytical balance (AG204 DeltaRange, Mettler Toledo, Ohio, USA). Both OD and CDW measurements were performed at least in a duplication.

### Custom-made cylindrical cage filled with acrylic fibres

**Fig. 8:**
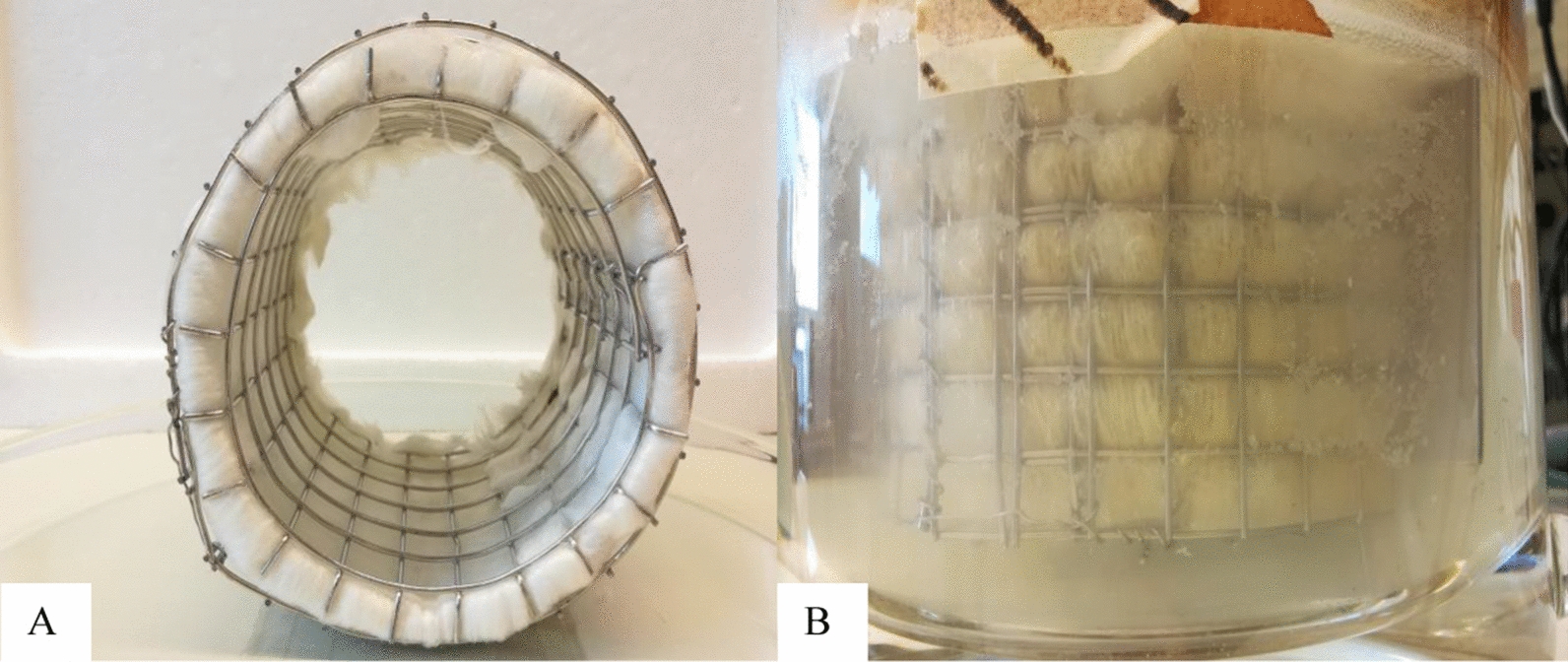
A custom-made cylindrical stainless-steel cage. **A** Preparation of a custom-made cylindrical stainless-steel cage equipped with acrylic fibres. **B** Installation of a custom-made cylindrical stainless-steel cage equipped with acrylic fibres in different continuous cultures performing in a continuous stirred tank reactor (CSTR)

Acrylic fibres were purchased from World of Wool (West Yorkshire, UK). A custom-made concentric cylindrical cage was made of stainless-steel mesh with the size of 1 mm × 1 mm × 0.4 mm. The cage was designed by three sizes with respect to an inner radius of 7.3 cm, a middle of radius 8.3 cm, and outer radius of 9.3 cm, which was for locking acrylic fibres in place, and was completely submerged in the culture (Fig. 8A). The cage equipped with acrylic fibres was autoclaved in water prior to assemblage inside the space between the baffles (Fig. 8B) and then autoclaved again with the culture medium. The sampling port, temperature port, and effluent port were placed inside the inner radius, whereas the pH probe and a biofilm sample port were set outside the cage.

### Preparation of chitosan

Chitosan (medium molecular weight, Sigma-Aldrich) was prepared as described earlier [37]. Briefly, 1 g of chitosan was mixed with 20 mL of 0.1 M HCl, followed by the addition of 5 mL of 50 mM Tris solution. pH of the chitosan solution was adjusted to pH 6 by adding 10 M NaOH. The final volume was 100 mL.

### Population dynamics

Genomic DNA of cells, as obtained from biofilms, were extracted by phenol–chloroform DNA extraction method, whereas GeneJET Genomic DNA purification kit (Thermo Fisher Scientific, USA) was used for planktonic samples for all culture conditions. DreamTaq DNA polymerase (Thermo Fisher Scientific) and EvaGreen^®^ (Biotium, Fremont, CA, USA) were added to the PCR master mix containing specific forward and reverse primers (Table 2) before loading to the PCR tubes (Bio-Rad, USA). The PCR protocol was carried out as follows: initial denaturation for 7 min at 95 °C, 32 cycles of denaturation for 30 s at 95 °C, annealing for 30 s at 61 °C with an extension of 20 s at 72 °C and this was repeated for 32 cycles. The melt curve was determined. Moreover, the resultant Cq values were evaluated to a standard curve generating from varying concentration of the genomic DNA of pure cultures. The genomic DNA of an individual species was used to obtain the initial quantity of DNA. The qPCR reactions were carried out in a Bio-Rad CXF96, for which the calibration curve and quantification of copy numbers were done by the software furnished by the qPCR instrument manufacturer (Bio-Rad, USA).

**Table 2:** Specific primers for qPCR analysis to determine the population dynamics

Target	Primer	Sequence	Product size (bp)
*C. kronotskyensis*	Ckron_F	CAGGAGATGGAACGTGGATT	224
	Ckron_R	CCATGGAGCAGTCCCACTAT	
*C*. *owensensis*	Cowen_F	GGCAAGTGGGAAGAAGATGA	190
	Cowen_R	CTCCGCAAGACTTGAACACA	

### Analytical methods for c-di-GMP

C-di-GMP was extracted as per the protocol described in [38] but some steps were modified. Briefly, 5 mL of culture medium was centrifuged at 4 °C using 4000 rpm in a swinging bucket rotor (Eppendorf 5810R, Eppendorf, Germany). The supernatant was discarded, followed by adding 300 µL of extraction solvent with cXMP (1 mg/mL) to the cell pellet. The samples were incubated on ice for 15 min and heated at 95 °C for 10 min. The samples were again incubated on ice for 5 min and centrifuged at 15,000 rpm for 8 min. The supernatant was collected into a new microcentrifuge tube. Finally, the pellet was washed twice with 200 µL of extraction solvent without cXMP. Only the supernatant was dried in the incubator at 50 °C overnight. The c-di-GMP extraction was performed in triplicates.

The quantification of c-di-GMP was measured as described earlier [16]. Briefly, the samples were separated in an Accela Ultra HPLC (Thermo Fisher Scientific, USA) equipped with a Kinetex XB-C18 column (2.1 × 50 mm, Phenomenex), using isocratic conditions, 2.5% MeOH (A) and 97.5% 10 mM ammonium acetate in 0.1% acetic acid (B) at the flow rate of 0.4 mL min^−1^ for 1.8 min. The internal standard of xanthosine 5ʹ-monophosphate (XMP) and c-di-GMP eluted at 0.8 min and 1.1 min, respectively. Standards were prepared with seven different concentrations ranging from 10 nM to10 µM.

The liquid samples were ionized in positive mode by electrospray ionization (ESI) on an Orbitrap Velos Pro mass spectrometer (Thermo Fisher Scientific, Waltham, USA). The signal was obtained with three scan events: (i) Full scan orbitrap (FTMS) (ii) and (iii) ion trap (ITMS) for quantification and multiple reaction monitoring (MRM) of XMP at m/z 347/153 and c-di-GMP at m/z 691/540.

## Supplementary Information

**Additional file 1: Table S1.** The concentration of substrates (glucose and xylose) in feed-in media, effluence, and substrate consumption rates at the dilution rates of 0.1–0.3 h^-1^. **Table S2.** Comparison of volumetric hydrogen productivity (Q_H2_) accomplished in the current study with previous studies. **Table S3.** The time interval of steady state between the dilution rates of 0.1–0.3 h^-1^ (Cases A–L).

## Data Availability

The datasets during and/or analysed during the current study available from the corresponding author on reasonable request.
