# Supplementary material for: Immobilization techniques improve volumetric hydrogen productivity of Caldicellulosiruptor species in a modified continuous stirred tank reactor
Source: Biotechnol Biofuels Bioprod. 2023 Feb 16;16:25. doi: 10.1186/s13068-023-02273-8 (PMC9933333; doi:10.1186/s13068-023-02273-8)
Supplement: Supplementary file 1 — Additional file 1: Table S1. The concentration of substrates (glucose and xylose) in feed-in media, effluence, and substrate consumption rates at the dilution rates of 0.1–0.3 h-1. Table S2. Comparison of volumetric hydrogen productivity (QH2) accomplished in the current study with previous studies. Table S3. The time interval of steady state between the dilution rates of 0.1–0.3 h-1 (Cases A–L). [file 13068_2023_2273_MOESM1_ESM.docx]

**Additional file**

**Table S1** The concentration of substrates (glucose and xylose) in feed-in media, effluence, and substrate consumption rates at the dilution rates between 0.1-0.3 h^-1^.

| **Conditions** | **Dilution rate (h^-1^)** | **Substrates  (mmol⋅L^-1^)** | | **Effluences (mmol⋅L^-1^)** | | | | | | **Substrate consumption rate**  **(mmol⋅L^-1^⋅h^-1^)** |
| --- | --- | --- | --- | --- | --- | --- | --- | --- | --- | --- |
|  |  | **Glucose** | **Xylose** | **Glucose** | **Xylose** | **Acetate** | **Lactate** | **Ethanol** | **Propionate** |  |
| Case A (*C.kronotskyensis* without carriers) | 0.1 | 41.02 ± 0.2 | 24.42 ± 0.3 | 22.62 ± 0.5 | 4.2 ± 0.9 | 30.23 ± 7.2 | 0.07 ± 0.1 | 0 | 0 | 3.82 ± 0.6 |
|  | 0.2 | 41.82 ± 0.1 | 22.71 ± 0.1 | 28.06 ± 0.5 | 7.97 ± 0.2 | 27.85 ± 0.2 | 0.09 ± 0 | 0 | 0 | 5.70 ± 0.1 |
|  | 0.3 | 40.75 ± 0 | 23.45 ± 0 | 33.31 ± 0.1 | 12.94 ± 0 | 17.39 ± 0.8 | 0 | 0 | 0 | 5.39 ± 0.3 |
| Case B (*C.kronotskyensis* with acrylic fibre) | 0.1 | 41.54 ± 0 | 21.46 ± 0.1 | 25.64 ± 1.1 | 4.19 ± 0.1 | 31.15 ± 2.0 | 1.64 ± 1.4 | 0 | 0 | 3.32 ± 0.2 |
|  | 0.2 | 41.18 ± 0.2 | 21.60 ± 0.3 | 21.20 ± 0.3 | 2.50 ± 0.1 | 35.54 ± 1.9 | 2.45 ± 0.3 | 0.67 ± 0.2 | 0.05 ± 0.1 | 7.82 ± 0.2 |
|  | 0.3 | 41.22 ± 0 | 21.69 ± 0.1 | 20.51 ± 0.2 | 6.40 ± 0.6 | 30.52 ± 0.9 | 6.78 ± 3.6 | 0.62 ± 0.2 | 0.40 ± 0.4 | 10.80 ± 0.3 |
| Case C (*C.kronotskyensis* with acrylic fibre and chitosan) | 0.1 | 41.35 ± 0 | 21.08 ± 0 | 22.04 ± 0.5 | 3.15 ± 0.5 | 49.64 ± 2.7 | 9.88 ± 0.3 | 0.93 ± 0.2 | 0.58 ± 0 | 3.80 ± 0.1 |
|  | 0.2 | 42.10 ± 0 | 22.03 ± 0.1 | 15.38 ± 0.1 | 3.06 ± 0.1 | 41.51 ± 1.9 | 13.76 ± 2.5 | 0.60 ± 0.3 | 0.30 ± 0.4 | 9.14 ± 0.3 |
|  | 0.3 | 41.33 ± 0 | 21.53 ± 0 | 17.83 ± 0.4 | 4.42 ± 0.2 | 37.80 ± 1.2 | 18.98 ± 0.4 | 0.30 ± 0.4 | 0.39 ± 0.1 | 12.20 ± 0.6 |
| Case D (*C.kronotskyensis* with chitosan) | 0.1 | 41.36 ± 0 | 21.67 ± 0 | 9.11 ± 1.4 | 1.11 ± 0.7 | 46.20 ± 4.9 | 1.48 ± 0.6 | 0 | 0 | 5.28 ± 0.2 |
|  | 0.2 | 41.46 ± 0 | 21.57 ± 0 | 20.91 ± 1.1 | 4.74 ± 0.2 | 22.89 ± 1.8 | 0.83 ± 0.1 | 0 | 0 | 7.48 ± 0.6 |
|  | 0.3 | 41.16 ± 0 | 21.25 ± 0 | 29.17 ± 0.3 | 12.17 ± 0.5 | 18.50 ± 0.2 | 0 | 0 | 0 | 6.32 ± 0.1 |
| Case E (*C. owensensis* without carriers) | 0.1 | N.A. | N.A. | N.A. | N.A. | N.A. | N.A. | N.A. | N.A. | N.A. |
|  | 0.2 | N.A. | N.A. | N.A. | N.A. | N.A. | N.A. | N.A. | N.A. | N.A. |
|  | 0.3 | N.A. | N.A. | N.A. | N.A. | N.A. | N.A. | N.A. | N.A. | N.A. |
| Case F (*C. owensensis* with acrylic fibre) | 0.1 | 41.21 ± 0 | 21.46 ± 0 | 6.13 ± 0.6 | 0.90 ± 0.4 | 39.65 ± 0.4 | 21.96 ± 0.4 | 0 | 0 | 5.56 ± 0 |
|  | 0.2 | 41.02 ± 0 | 22.33 ± 0 | 12.35 ± 0.5 | 1.97 ± 0.2 | 27.80 ± 0.4 | 23.38 ± 1.3 | 0 | 0 | 9.81 ± 0.2 |
|  | 0.3 | 41.30 ± 0 | 22.13 ± 0 | 12.32 ± 0.3 | 3.57 ± 0.1 | 22.38 ± 0.2 | 28.40 ± 4.2 | 0 | 0 | 14.78 ± 0.4 |
| Case G (*C. owensensis* with acrylic fibre and chitosan) | 0.1 | 41.39 ± 0 | 21.56 ± 0 | 4.70 ± 0.6 | 0.99 ± 0.4 | 46.06 ± 0.6 | 26.24 ± 1.1 | 0 | 0 | 5.73 ± 0 |
|  | 0.2 | 41.44 ± 0 | 21.14 ± 0 | 7.93 ± 0.1 | 1.21 ± 0.4 | 31.73 ± 1.7 | 28.63 ± 0 | 0 | 0 | 10.69 ± 0.3 |
|  | 0.3 | 41.72 ± 0.2 | 21.97 ± 0 | 8.10 ± 0.2 | 1.14 ± 0.3 | 27.14 ± 0.3 | 33.53 ± 3.2 | 0 | 0 | 16.33 ± 1.8 |
| Case H (*C. owensensis* with chitosan) | 0.1 | 41.42 ± 0 | 21.87 ± 0.3 | 22.15 ± 0 | 0 | 38.30 ± 0.1 | 0 | 0 | 0 | 4.11 ± 0 |
|  | 0.2 | 41.08 ± 0.1 | 21.11 ± 0.1 | 27.11 ± 0.5 | 1.62 ± 0.6 | 26.82 ± 1.3 | 1.68 ± 0.1 | 0 | 0 | 6.69 ± 0 |
|  | 0.3 | 40.44 ± 0.4 | 21.33 ± 0 | 27.02 ± 0.4 | 6.32 ± 0.2 | 18.62 ± 0.1 | 2.39 ± 0.3 | 0 | 0 | 8.53 ± 0.1 |
| Case I (Co-culture without carriers) | 0.1 | 41.48 ± 0 | 21.67 ± 0 | 19.24 ± 1.1 | 2.12 ± 0.4 | 32.39 ± 0 | 8.84 ± 0.4 | 0 | 0 | 4.18 ± 0 |
|  | 0.2 | 41.13 ± 0 | 21.13 ± 0.1 | 24.62 ± 0.7 | 6.56 ± 0.2 | 23.05 ± 0 | 1.21 ± 0.8 | 0 | 0 | 6.22 ± 0.3 |
|  | 0.3 | 41.01 ± 0 | 21.67 ± 0 | 29.69 ± 0.5 | 9.09 ± 0.6 | 14.73 ± 0.4 | 4.44 ± 1.5 | 0 | 0 | 7.08 ± 0.4 |
| Case J (Co-culture with acrylic fibre) | 0.1 | 41.52 ± 0 | 21.04 ± 0 | 6.08 ± 0.7 | 1.05 ± 0.4 | 41.61 ± 0.7 | 22.84 ± 0.8 | 4.72 ± 5.2 | 0 | 5.54 ± 0.1 |
|  | 0.2 | 40.66 ± 0 | 21.11 ± 0 | 9.17 ± 0.3 | 3.08 ± 0.4 | 27.17 ± 4.3 | 21.76 ± 4.7 | 6.88 ± 1.1 | 0 | 9.90 ± 0.2 |
|  | 0.3 | 41.53 ± 0 | 20.77 ± 0 | 12.85 ± 0.8 | 3.11 ± 0.7 | 22.38 ± 0.7 | 25.62 ± 0.6 | 3.01 ± 0.8 | 1.43 ± 0 | 13.90 ± 0.2 |
| Case K (Co-culture with acrylic fibre and chitosan) | 0.1 | 41.12 ± 0 | 21.71 ± 0 | 6.04 ± 0.3 | 0.93 ± 0.3 | 41.61 ± 0.7 | 22.84 ± 0.8 | 4.05 ± 0.1 | 1.01 ± 0.3 | 5.59 ± 0.1 |
|  | 0.2 | 41.47 ± 0 | 21.01 ± 0 | 6.47 ± 0.4 | 1.91 ± 0.8 | 27.17 ± 4.3 | 21.76 ± 4.7 | 1.54 ± 2.2 | 0.46 ± 0.8 | 10.82 ± 0.2 |
|  | 0.3 | 41.52 ± 0.1 | 21.06 ± 0 | 8.90. ± 0.7 | 2.43 ± 0.1 | 22.38 ± 0.7 | 25.62 ± 0.6 | 5.96 ± 4.6 | 0.96 ± 0.9 | 15.37 ± 0.2 |
| Case L (Co-culture with chitosan) | 0.1 | 40.67 ± 0 | 20.58 ± 0 | 27.51 ± 0.3 | 7.53 ± 0.6 | 28.96 ± 3.0 | 4.58 ± 0.4 | 0 | 0 | 2.62 ± 0.3 |
|  | 0.2 | 41.44 ± 0 | 20.17 ± 0 | 31.52 ± 0.5 | 8.64 ± 1.0 | 23.79 ± 1.6 | 0.71 ± 0.2 | 0 | 0 | 4.29 ± 0.5 |
|  | 0.3 | 40.83 ± 0 | 21.57 ± 0 | 26.49 ± 0.7 | 8.51 ± 2.9 | 14.91 ± 2.9 | 0.80 ± 0.1 | 0 | 0 | 8.22 ± 2.8 |

* N.A. means data not available

**Table S2** Comparison of volumetric hydrogen productivity (Q_H2_) accomplished in the current study with previous studies.

(CSTR = continuous stirred tank reactor, UA = anaerobic upflow reactor)

| **Organism** | **Sugar concentration**  **(g⋅L^-1^)** | **Reactor type** | **Fermentation mode** | **Carrier** | **Hydrogen productivity (mmol⋅L^-1^⋅h^-1^)** | **Reference** |
| --- | --- | --- | --- | --- | --- | --- |
| ***C*. *saccharolyticus*** | Glucose: 4.4 | CSTR | Continuous | Not stated | 12.4 | de Vrije et al. (2007) |
| **Thermophilic culture (33HL)** | Glucose: 6 | CSTR | Continuous | Polymeric carriers | 45.8 | Koskinen et al. (2008) |
| ***C*. *saccharolyticus*** | Glucose: 5.4 | Trickle bed reactor | Continuous | Not stated | 22 | Groenestijn et al. (2009) |
| ***C*. *saccharolyticus*** | Glucose: 5  Xylose: 5 | CSTR | Continuous | Not stated | 11.6 | Zeidan et al. (2010) |
| ***C*. *saccharolyticus***  ***C*. *owensensis*** | Glucose: 10 | UA | Continuous  *D* = 1.25 h^-1^ | Granular sludge | 20 | Pawar et al. (2015) |
| ***C*. *saccharolyticus***  ***C*. *owensensis*** | Glucose: 10 | CSTR | Continuous  *D* = 0.1 h^-1^ | K-1 carrier | 8 | Pawar et al. (2015) |
| ***C*. *saccharolyticus***  ***C*. *owensensis*** | Glucose: 10 | CSTR | Continuous  *D* = 0.8 h^-1^ | Chitosan | 8.4 | Vongkampang et al. (2021) |
| ***C*. *kronotskyensis*** | Gglucose: 7.3  Xylose: 3.4 | CSTR | Continuous  *D* = 0.3 h^-1^ | Chitosan and acrylic fibre | 30±0.2 | **This study (Case C)** |
| ***C*. *kronotskyensis***  ***C*. *owensensis*** | Glucose: 7.3  Xylose: 3.4 | CSTR | Continuous  *D* = 0.3 h^-1^ | Acrylic fibre | 26.4±1.9 | **This study (Case J)** |
| ***C*. *kronotskyensis*** | Gglucose: 7.3  Xylose: 3.4 | CSTR | Continuous  *D* = 0.3 h^-1^ | Acrylic fibre | 25.4±0.6 | **This study (Case B)** |

**Table S3** The time interval of steady state between the dilution rate of 0.1-0.3 h^-1^ (Case A-L).

| **Conditions** | **Dilution rate (h^-1^)** | **Steady state (h)** | |
| --- | --- | --- | --- |
|  |  | **1^st^** | **2^nd^** |
| Case A (*C.kronotskyensis* without carriers) | 0.1 | 149 | 172 |
|  | 0.2 | 218 | 244 |
|  | 0.3 | 365 | 390 |
| Case B (*C.kronotskyensis* with acrylic fibre) | 0.1 | 149 | 172 |
|  | 0.2 | 219 | 244 |
|  | 0.3 | 373 | 390 |
| Case C (*C.kronotskyensis* with acrylic fibre and chitosan) | 0.1 | 220 | 265 |
|  | 0.2 | 315 | 364 |
|  | 0.3 | 408 | 414 |
| Case D (*C.kronotskyensis* with chitosan) | 0.1 | 266 | 291 |
|  | 0.2 | 408 | 433 |
|  | 0.3 | 468 | 483 |
| Case E (*C. owensensis* without carriers) | 0.1 | N.A. | N.A. |
|  | 0.2 | N.A. | N.A. |
|  | 0.3 | N.A. | N.A. |
| Case F (*C. owensensis* with acrylic fibre) | 0.1 | 143 | 167 |
|  | 0.2 | 220 | 240 |
|  | 0.3 | 295 | 323 |
| Case G (*C. owensensis* with acrylic fibre and chitosan) | 0.1 | 144 | 167 |
|  | 0.2 | 220 | 240 |
|  | 0.3 | 309 | 335 |
| Case H (*C. owensensis* with chitosan) | 0.1 | 267 | 293 |
|  | 0.2 | 457 | 481 |
|  | 0.3 | 658 | 676 |
| Case I (Co-culture without carriers) | 0.1 | 84 | 97 |
|  | 0.2 | 144 | 168 |
|  | 0.3 | 219 | 243 |
| Case J (Co-culture with acrylic fibre) | 0.1 | 149 | 173 |
|  | 0.2 | 432 | 461 |
|  | 0.3 | 526 | 549 |
| Case K (Co-culture with acrylic fibre and chitosan) | 0.1 | 119 | 169 |
|  | 0.2 | 338 | 372 |
|  | 0.3 | 411 | 486 |
| Case L (Co-culture with chitosan) | 0.1 | 149 | 170 |
|  | 0.2 | 245 | 267 |
|  | 0.3 | 365 | 390 |

* N.A. means data not available
